# Mexican-National Institute of Neurology and Neurosurgery-Stroke Registry: Results of a 25-Year Hospital-Based Study

**DOI:** 10.3389/fneur.2018.00207

**Published:** 2018-04-04

**Authors:** Antonio Arauz, Juan Manuel Marquez-Romero, Miguel A. Barboza, Fabiola Serrano, Carol Artigas, Luis Manuel Murillo-Bonilla, Carlos Cantú-Brito, José Luis Ruiz-Sandoval, Fernando Barinagarrementeria

**Affiliations:** ^1^Stroke Clinic, Instituto Nacional de Neurología y Neurocirugía, Mexico City, Mexico; ^2^Instituto Mexicano del Seguro Social (IMSS) HGZ 2, Aguascalientes, Mexico; ^3^Facultad de Medicina Unidad Autónoma de Guadalajara, Guadalajara, Mexico; ^4^Instituto Nacional de Ciencias Médicas y Nutrición Salvador Zubirán, Mexico City, Mexico; ^5^Hospital Civil de Guadalajara, Guadalajara, Mexico; ^6^Universidad del Valle de México, Querétaro, Mexico

**Keywords:** stroke classification, vascular risk factors, stroke outcome, stroke registry, Mexico

## Abstract

**Background and purpose:**

Stroke has been scarcely studied in Latin America (LA). The Mexican Institute of Neurology Stroke Registry was established in 1990 as a prospective computer-based database to register data obtained from patients admitted with stroke. Using this data, we attempted to define the profile of risk factors and outcomes.

**Methods:**

The demographic data, stroke description, ancillary tests, vascular risk factors, and modified Rankin scale (mRs) were registered. Ischemic stroke subtyping was based on the Trial of Org 10,172 of the Acute Stroke Treatment classification. We followed-up patients using multiple overlapping methods. Primary outcomes included mRs, recurrence, and death at 30 days and at the end of follow-up.

**Results:**

We included 4,481 patients with a median follow-up of 27 months, (17,281 person-years follow-up). The mean age was 52.8 ± 18 years. There were 2,229 males (50%) included in the study. CI was present in 64.9%, intracerebral hemorrhage (ICH) in 25.6%, and cerebral venous thrombosis (CVT) in 6.3%. Hypertension was the major risk factor (46.5%). The most common cause of CI was atherosclerosis (27%). ICH was mainly hypertensive (58%), and 60% of CVT were puerperal. Overall, the mortality rate was 24.5%. The recurrence rate was 16.9%. Poor outcome (mRs ≥ 3) was found in 56.2% of patients. The best outcomes were observed in CVT patients (74.5% mRs ≤ 2), whereas 72.1% ICH patients had mRs ≥3.

**Conclusion:**

This is one of the largest hospital-based registries in LA and shows significant differences with other previously published registries, including a younger age, relatively less hypertension, and larger proportion of CVT. Poor functional outcome was common. This study adds to the understanding of geographic differences in stroke characteristics and outcomes.

## Introduction

Reports from various stroke registries have provided wide and extensive analyses of incidence, risk factors, and mortality (1–4). The relative importance of different stroke risk factors may differ among countries, and there are recognized geographic variations in the incidence of and mortality from stroke. However, no large hospital-based stroke registries have been published from Mexico, and stroke has been scarcely studied in Latin America (LA).

Mexico has a heterogeneous population, with different environment, lifelong dietary patterns, and other habits, which result in an increased prevalence of vascular risk factors and chronic diseases that play a role in the pathogenesis of stroke (5).

The National Institute of Neurology and Neurosurgery-Stroke Registry (NINN-SR) was established in 1990 as a prospective database to register data from all patients admitted with stroke. Using these data, we attempted to estimate the type and severity of stroke as well as define the profile of risk factors and outcomes.

## Materials and Methods

The NINN-SR is a prospective observational stroke databank gathered by the Stroke Clinic of the National Institute of Neurology and Neurosurgery in Mexico City, over a period from January 1990 to January 2015. The hospital is a 111-bed academic referral hospital that serves an adult population over 15 years. Most of the NINN admitted patients come from Mexico City area, which covers a 1,485 km^2^ with nearly 20.4 million urban inhabitants (for the Greater Mexico City), with few patients from the peripheral areas or other Mexican States. The NINN is a public-founded institution, part of the Mexican Health Secretary Department, and provides neurologic medical assistance to low- and middle-income population referred from hospitals all around the country.

### Setting and Population

We prospectively recruited consecutive patients admitted to or seen as outpatients at our hospital. In this study, we included patients with a first clinical stroke, as demonstrated by brain imaging. All ischemic strokes (IS), primary intracerebral hemorrhages (ICHs), and cerebral venous thrombosis (CVT) admitted as an “acute stroke” case or a subacute/chronic clinical syndrome. Since the cases of subarachnoid hemorrhage in our hospital are treated in the neurosurgery department, they were excluded from this registry.

Stroke subtype classification was based on the modified Trial of Org 10,172 of the Acute Stroke Treatment (TOAST) classification using a consensus approach (6) and modified if necessary by the findings of subsequent ancillary diagnostic tests. We followed-up patients for as long as they were available using multiple overlapping methods, including regular patient visits to the clinic (4,556 patients), telephonic contact with the patient or patients’ relatives (1,074 patients), and review of hospital medical records (5,630 patients), and the hospital death register (for in-hospital death cases). In cases of death during follow-up, we attempted to review all relevant medical records to confirm the cause of death or at least locate the death certificate (together with telephonic information from patients’ relatives).

### Data Recorded

Data included 156 items each with 2–10 possible values that were coded and entered in a computerized databank by one of the authors. *Baseline characteristics*: age, sex, and living conditions (rural or urban). *Vascular risk factors and comorbid conditions*: hypertension (previous diagnosis, current treatment or values ≥140/90 mmHg in at least two subsequent measurements), atrial fibrillation (AF) [history of chronic AF, supported by past electrocardiogram (ECG)] and positive ECG during hospitalization, or past medical history with positive ECG, previous myocardial infarction (previous diagnosis based on a documented transient elevation of biochemical markers of myocardial necrosis with typical ECG signs), transient ischemic attack [(TIA) acute neurological deficit of vascular origin, lasting <24 h], diabetes mellitus (previous diagnosis, concurrent treatment with insulin or oral hypoglycemic medications, or fasting plasma glucose level ≥140 mg/dL), smoking [current or former practice (subject had stopped smoking for at least 2 years preceding the stroke event)], history of migraine, venous hematocrit of >50%, and family history of stroke or acute myocardial infarction.

### Clinical and Paraclinical Assessment

In addition to cerebral computed tomography (CT) and/or magnetic resonance (MR) imaging, all patients received a standardized workup to rule out definite causes of stroke. The workup consisted of routine blood tests and a coagulation study (tests for protein S, protein C, antithrombin III, and anti-phospholipid antibodies were performed only in young patients with no other vascular risk factors), 12-lead ECG and echocardiography, and at least one of the following vascular studies performed within 3 months after the onset of stroke: catheter angiography, MR angiography (MRA), CT angiography (CTA), and cervical and Transcranial Doppler.

The following disorders were considered to be definite causes of IS: large-artery atherosclerosis (defined by a stenosis of at least 50% or occlusion of the corresponding vessel); lacunar stroke (defined by a small, deep infarct less than 15 mm in diameter in a patient with hypertension); cardioembolic causes, such as AF, recent (within 4 months before the stroke) myocardial infarction, dilated cardiomyopathy, rheumatic mitral stenosis, mitral or aortic vegetations or prostheses, left atrial or left ventricular thrombus or tumor, akinetic left ventricular segment, spontaneous echo contrast of the left atrium, and complex atheroma of the aortic arch, and other determined etiologies (ODE) of stroke, such as nonatherosclerotic arteriopathies (e.g., dissection), coagulopathies, hematologic or systemic disorders (e.g., the anti-phospholipid-antibody syndrome). Unidentified etiology with negative evaluation (UE-NE) was recorded in patients with no likely etiology determined despite an extensive evaluation. In the cases when the cause of a stroke could not be determined with confidence due to insufficient ancillary diagnostic tests, unidentified etiology with incomplete evaluation was recorded; on the other hand, cases with two or more possible etiologies or disagreement among raters, were reviewed by other two stroke neurologists from the Stroke Clinic, to determine according to the level of evidence of the studies, the more suitable etiology.

For each patient, the clinical, laboratory, and imaging data were reviewed by at least two of the authors.

Because of the lack of treatment influence, the study was exempt from informed consent. However, patients or family members if the patient was neurologically impaired, verbally agreed to participate in the project. This study was approved by the Institutional Ethics Committee.

### Outcome Measures

The following outcome events were systematically recorded: functional outcome, as measured by the modified Rankin scale (mRs), at 30 days or at discharge, whichever occurred first, 1 year and end of individual follow-up; recurrent stroke, as defined by the acute occurrence of focal neurological signs lasting for more than 24 h, in a different location from that of the previous stroke or worsening of an existing deficit that lasted for more than 1 week or more than 24 h if accompanied by a new lesion on neuroimaging. Good clinical outcome was defined as a mRs ≤2. Only patients that had a regular follow-up visit at the Stroke Clinic and complete data at their medical records were included for outcome events analysis.

### Statistical Analysis

For statistical analysis, the SPSS 22.0 package (IBM SPSS Statistics for Windows, Armonk, NY, USA: IBM Corp.) was used. Comparisons between groups were analyzed using the χ^2^ test, Fisher’s exact test, *t*-test for unpaired data, or analysis of variance, as appropriate; normality tests for continuous variables were applied. Potential risk factors for recurrent and fatal events were identified by logistic-regression analysis. Kaplan–Meier survival analysis was used to assess the absolute risk of recurrent cerebrovascular events; patients lost during the first year follow-up were censored from the analysis. Functional status was divided into good functional status (mRs 0–2) and bad functional status (mRs 3–6), and univariate analysis was performed to determine the impact of each independent variable, with the subsequent selection for multivariate analysis in the acute phase of the index stroke (at discharge). The predictive value for each category of stroke with respect to recurrent and fatal cerebrovascular events was assessed using log-rank tests and Cox proportional hazards models. All tests were two-tailed. Covariates included for multivariate analysis were selected according to the association risk classified as significant (*p* < 0.1), and were included according with predefined risk association status [age, gender, risk factors, category of the infarction (according to NIH stroke scale), and functional status (mRs)]. Significance was defined as *p* < 0.05 and also expressed as odds ratios (OR) and the 95% confidence interval.

## Results

### Patients and Follow-Up

We recruited and registered data obtained from 5,630 patients, and 261 (4.6%) of these patients were excluded due to the absence of subsequent registry (25 cases), lack of initial neuroimaging (69 cases), or follow-up (120 cases). Thus, only 5,369 patients with a brain imaging confirmed first stroke with a follow-up of ≥3 months were included in the analysis. Patients were followed for a median of 23 (IQR, 6–58) months, giving 18,437 person years of follow-up time, with all patients followed-up using at least 1 of our methods.

There were 2,652 males (mean age 53.6 ± 17.2 years) and 2,717 females (50.6%) with a mean age of 51.6 ± 19.6 years. IS was present in 3,509 patients (65.3%), TIA in 165 patients (3.1%), ICH in 1,352 patients (25.1%), and CVT in 343 patients (6.4%).

The median duration of hospitalization was 9 (IQR 2–16) days. Overall, 5,062 patients (94.3%) received a CT scan of the brain during the initial study, and an initial study was performed in 307 patients using MR. Subsequent MR (from the first CT scan) was performed in 3,633 patients (67.6%). Vasculature was examined in 2,708 (50.4%) patients with a cervical ultrasound, in 616 patients (11.4%) with CTA, and in 984 patients (18.3%) with MRA. Catheter angiography was performed in 1,951 patients (36.3%; the NINN is a third level referral center, with one of the first endovascular laboratories in Mexico, therefore, previous to angio-MR many cases were transferred for selective angiography to confirm etiology). In total, 1,852 patients (34.5%) underwent transthoracic echocardiography. Transesophageal echocardiography was performed in 712 patients (14.3%). Prothrombotic serum proteins and anti-phospholipids antibodies were determined in 696 patients (12.9%).

### Vascular Risk Factors

A previous medical history of hypertension was found in 46.6% of all stroke-type patients. The prevalence of hypertension was higher in patients with ICH compared with other types of stroke (*p* < 0.05). All of the vascular risk factors were observed less frequently, as expected, in CVT patients (*p* < 0.001) and in the UE-IE stroke subtype (*p* < 0.001).

### Ischemic Stroke

Affected vascular territories were distributed as follows; carotid artery territory infarct accounted for 67.9% of cases (2,494 patients), vertebrobasilar infarct was present in 31.2% of patients, and watershed infarcts were present in 0.9% of patients. Table 1 provides more detailed information on the characteristics of IS patients according to their functional status at discharge.

**Table 1:** General features and vascular risk factors according to clinical outcome at discharge modified Rankin scale (mRs) in patients with ischemic stroke.

	Good clinical outcome (mRs 0–2) *n* = 1,484 (%)	Bad clinical outcome (mRs 3–6) *n* = 2,025 (%)	Total*N* = 3,509 (%)	*p*
Age, years^a^	55 (42–68)	60 (46–72)	57 (41–69)	<0.001*
NIHSS (admission)	4 (2–8)	14 (8–19)	10 (5–16)	<0.001*
**Risk factors**				
Hypertension	643 (43.3)	989 (48.8)	1,632 (46.5)	0.001
Diabetes mellitus	320 (21.6)	479 (23.7)	799 (22.8)	0.14
Current smoking	390 (26.3)	518 (25.6)	908 (25.9)	0.64
Coronary disease	94 (6.3)	163 (8.0)	257 (7.3)	<0.05
Hypercholesterolemia	312 (21.0)	309 (15.3)	621 (17.7)	0.001
Known atrial fibrillation	100 (5.5)	166 (9.8)	266 (7.6)	<0.001
**Therapeutic approach**				
Antiplatelet therapy	1,161 (78.2)	1,463 (72.2)	2,624 (74.8)	<0.001
Anticoagulation	290 (19.5)	473 (23.4)	763 (21.7)	0.007
Statin	237 (15.1)	256 (12.6)	493 (14.0)	0.005
Thrombolysis	30 (2.0)	40 (2.0)	70 (2.0)	0.92
Decompressive cranectomy	18 (1.2)	106 (5.2)	124 (3.5)	<0.001
**Etiology**				
Large-artery atherosclerosis	323 (21.8)	558 (27.6)	881 (25.1)	<0.001
Small vessel disease	288 (19.4)	242 (12.0)	530 (15.1)	<0.001
Cardioembolism	315 (21.2)	546 (27.0)	861 (24.5)	<0.001
Other conditions^b^	260 (17.5)	337 (16.6)	597 (17.0)	0.50
Cryptogenic	129 (8.7)	97 (4.8)	226 (6.4)	<0.001
UE-IE	158 (10.6)	235 (11.6)	393 (11.2)	0.37

*^a^Median (interquartile range)*.

*p-Value by U-Mann–Whitney Wilcoxon.

*^b^Other causes: nonatherosclerotic arteriopathies (dissection, vasculitis, migraine, vasospasm, dolichoectasia, acquired, or hereditary thrombophilia)*.

*UE-IE, undetermined etiology-incomplete evaluation; mRs, modified Rankin score*.

Therapeutic approach of acute management in IS patients is detailed in Table 1. Only 2% of acute cases that were referred to the NINN Emergency Department fulfilled criteria for acute IV thrombolysis. Cardioembolic conditions accounted for 24.6% of all stroke causes, with nearly 88.6% of all of them with oral anticoagulation for secondary prevention.

### Intracerebral Hemorrhage

The most common location for ICH was the basal ganglia, 43.8% (lenticular 34.4%, thalamo-lenticular 32.5%, pure thalamic 29.0%, and caudate 4.1%); 467 patients (34.5%) had lobar ICH (frontal 37.2%, parietal 27.6%, temporal 18.3%, and occipital 16.9%). Posterior fossa ICH was diagnosed in 196 patients (14.5%) with the cerebellum indicated as the most commonly affected region (44.4%). There were 18 cases (1.3%) of pure intraventricular hemorrhage [although an intraventricular component of the hemorrhage was present in 463 (34.2%) of patients] and 19 cases (1.4%) of multiple hemorrhages. Table 2 describes detailed data according to their functional status at discharge.

**Table 2:** General features and vascular risk factors according to clinical outcome at discharge modified Rankin scale (mRs) in patients with intracerebral hemorrhage.

	Good clinical outcome (mRs 0–2)*n* = 392 (%)	Bad clinical outcome (mRs 3–6) *n* = 960 (%)	Total *N* = 1,352 (%)	*p*
Age, years^a^	42 (28–59)	55 (43–66)	53 (37–65)	<0.001*
**Risk factors**				
Hypertension	136 (34.7)	624 (65.0)	760 (56.2)	<0.001
Diabetes mellitus	35 (8.9)	176 (18.3)	211 (15.6)	<0.001
Current smoking	69 (17.6)	211 (22.0)	280 (20.7)	0.07
Hypercholesterolemia	43 (11.0)	89 (9.3)	132 (9.8)	0.34
**Therapeutic approach**				
Decompressive cranectomy	3 (0.8)	43 (4.5)	46 (3.4)	0.001
Intraventricular drainage	11 (2.8)	158 (16.5)	169 (12.5)	<0.001
Hematoma evacuation	32 (8.2)	99 (10.3)	131 (9.7)	0.22
Medical therapy only	289 (71.9)	559 (58.2)	841 (63.2)	<0.001
**Etiology**				
Hypertensive	129 (32.9)	637 (66.4)	766 (57.0)	<0.001
Amyloid angiopathy	12 (3.1)	23 (2.4)	35 (2.6)	0.48
AVM	74 (18.9)	76 (7.9)	150 (11.2)	<0.001
Cavernous malformation	60 (15.3)	42 (4.4)	102 (7.5)	<0.001
Drug related (anticoagulants/antiplatelet)	11 (2.8)	37 (3.9)	48 (3.6)	0.34
Idiopathic	44 (11.2)	41 (4.3)	85 (6.3)	<0.001

*^a^Median (interquartile range)*.

*p-Value by U-Mann–Whitney Wilcoxon.

*AVM, arterio-venous malformation; mRs, modified Rankin score*.

In total, 85.1% of 766 patients with hypertension-associated ICH had a previous diagnosis of hypertension, but 84.3% of patients had either suspended medication or were taking their anti-hypertensive medication irregularly. Only 153 patients (19.9%) did not know that they had arterial hypertension.

### Cerebral Venous Thrombosis

As shown in Table 3, nearly 83.9% of all CVT patients were females, with a mean age that was significantly minor (29 years) compared with other patients in the registry (*p* < 0.05). Pregnancy-related CVT (Pregnancy + puerperium) accounted for 179 (52.2%) of all CVT cases. A complete diagnostic work up was obtained, and no etiology could be determined; however, other etiologies were present in 35 cases (infection, dural fistula, severe dehydration). Catheter angiography was performed in 170 patients (49.5%), whereas 262 patients (76.4%) had MRA. Nevertheless, the diagnosis of CVT, which relied solely on the findings by MRA, was observed in only 117 patients. Most CVT (72.8%) produced venous infarcts that were observed bilaterally in 96 patients (27.9%). The hemorrhagic component of the venous infarct was observed in 132 patients (38.4%). Hematocrit was significantly lower among CVT patients compared with other stroke subtypes (*p* < 0.05), in which the mean hematocrit only was even lower in puerperal CVT (35.5 ± 7.0%, *p* < 0.01).

**Table 3:** General features and vascular risk factors according to clinical outcome at discharge modified Rankin scale (mRs) in patients with cerebral venous thrombosis.

	Good clinical outcome (mRs 0–2)*n* = 240 (%)	Bad clinical outcome (mRs 3–6) *n* = 103 (%)	Total *N* = 343 (%)	*p*
Age, years^a^	28 (22–37)	29 (23–40)	29 (22–38)	0.32
**Risk factors**				
Oral contraceptive	9 (8.7)	40 (16.7)	48 (14.1)	0.004
Pregnancy related	28 (11.8)	19 (18.4)	47 (13.9)	0.10
Puerperium	92 (38.5)	42 (40.8)	134 (39.2)	0.70
Current smoking	16 (15.5)	20 (8.3)	36 (10.5)	0.04
Previous VPT	15 (6.3)	4 (3.9)	19 (5.5)	0.38
Known acquired thrombophilia	7 (2.9)	2 (1.3)	9 (3.0)	0.27
**Therapeutic approach (discharge)**				0.35
Anticoagulation	159 (66.3)	85 (82.5)	244 (71.1)	0.002
Antiplatelet	81 (33.8)	18 (17.5)	99 (28.9)	0.002
**Etiology**				
Provoked CVT^b^	145 (60.4)	64 (62.1)	209 (60.9)	0.76
Prothrombotic conditions^c^	48 (20.0)	20 (19.4)	68 (19.8)	0.90
Unknown	33 (13.7)	33 (32.0)	66 (19.2)	0.001

*^a^Median (interquartile range)*.

*p-Value by U-Mann–Whitney Wilcoxon.

*^b^Provoked CVT: pregnancy and puerperium, oral contraceptives, drugs, parameningeal infections, systemic diseases, cancer related*.

*^c^Prothrombotic conditions: antithrombin III deficiency, protein C deficiency, protein S deficiency, anti-phospholipid syndrome, mutation G20210A of factor II, hyperhomocysteinemia*.

*VPT, venous peripheral thrombosis; CVT, cerebral venous thrombosis; mRs, modified Rankin score*.

### Outcome

**Figure 1:**
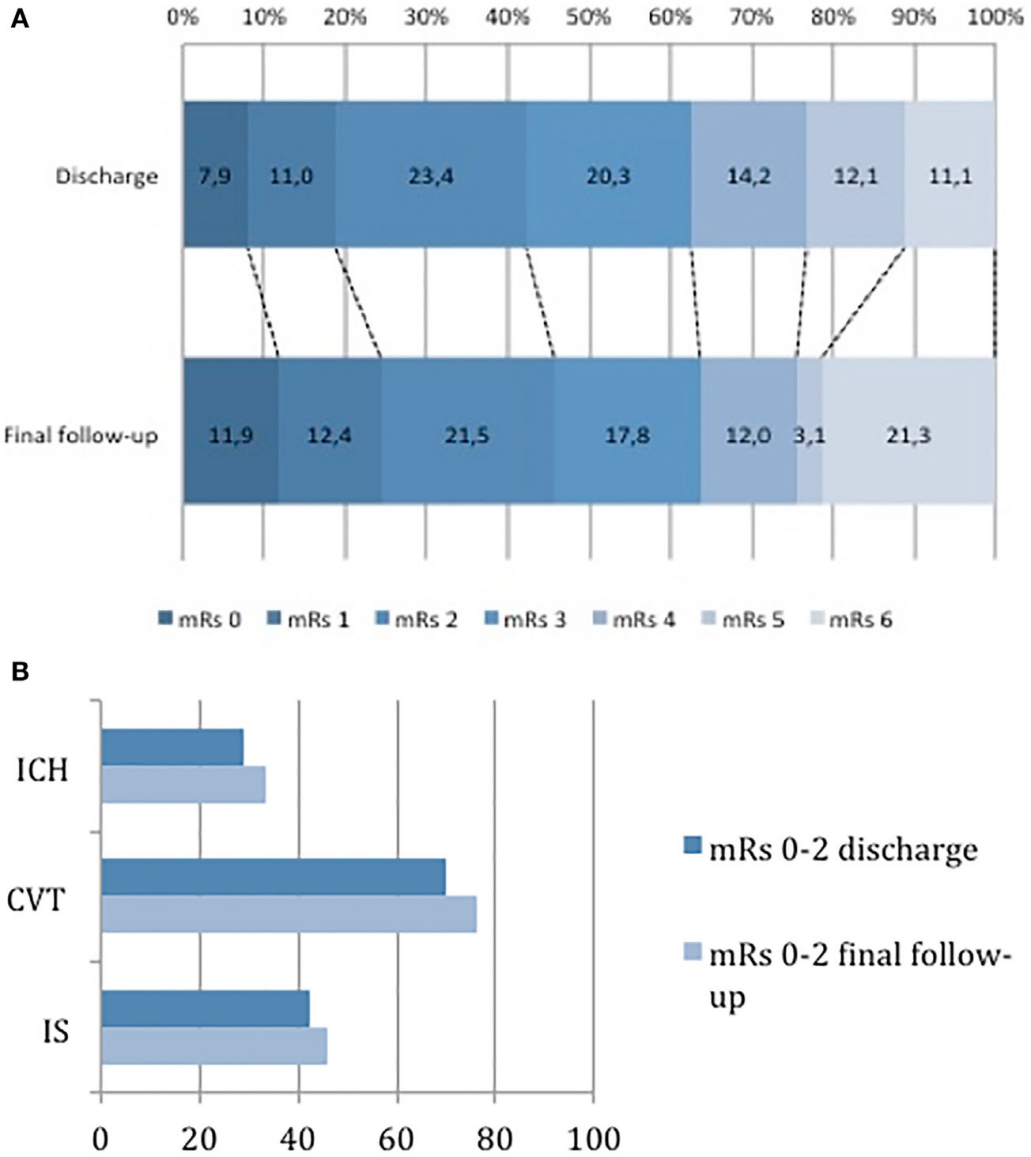
Functional outcome as measured by the modified Rankin scale in all patients **(A)**, and by stroke subtype **(B)**. Abbreviations: mRs, modified Rankin score; ICH, intracranial hemorrhage; CVT, cerebral venous thrombosis; IS, ischemic stroke.

The functional outcomes are shown in Figure 1, and can be seen on each subtype of stroke on Tables 1–3. Good clinical outcome was observed in 2,268 patients (40.3%) at 30 days/discharge, whereas 3,362 (59.7%) patients had a poor outcome. Mortality at 30 days/discharge was 11.1%. The best functional outcomes were observed in 70.0% of CVT patients with mRs ≤2, and mortality at 30 days/discharge occurred in 7.3% of CVT patients. However, the worst prognosis, including the highest mortality, corresponded to ICH patients, at 71.0% (960 patients), with mRs ≥3, of which 21.3% died.

**Figure 2:**
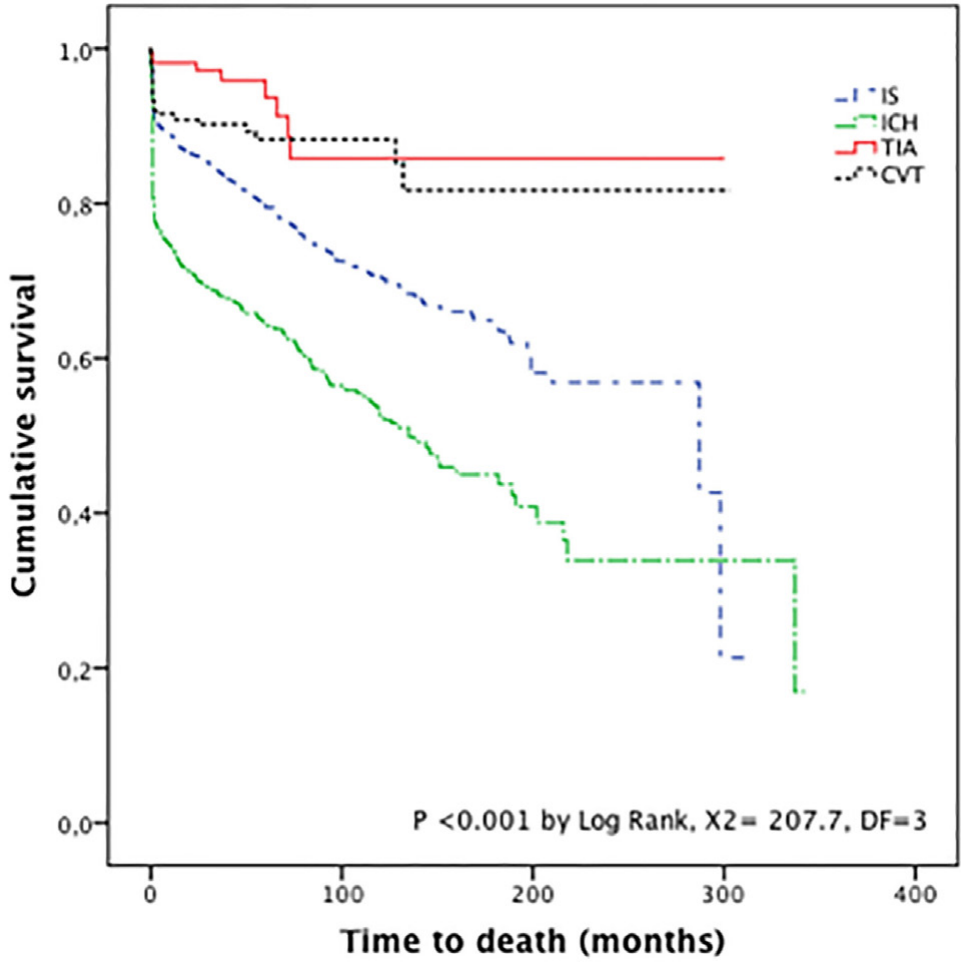
Kaplan–Meier survival curves by stroke subtype. Abbreviations: IS, ischemic stroke; ICH, intracranial hemorrhage; TIA, transient ischemic attack; CVT, cerebral venous thrombosis.

At the end of the follow-up, 2,460 patients (45.8%) had a good outcome, as shown in Figure 1. Figure 2 shows the Kaplan–Meier survival curves of the four stroke subtypes. Overall, mortality was 21.3% (1,145 deaths at the end of follow-up), of which 18.0% occurred in IS patients, 34.6% in ICH patients, and 10.2% in CVT patients. Log-rank testing of survival showed a significant difference between stroke subtypes; ICH vs. CVT: *p* < 0.001; ICH vs. IS: *p* < 0.05; and IS vs. CVT: *p* < 0.05. Among the survivors, having a bad functional outcome (mRs >2) at discharge was a strong predictor of death (HR 11.6, 95% CI 9.25–14.5, *p* < 0.001); which is consistent after covariate adjustment [age, sex, risk factors; HR 10.6 (95% CI 8.43–13.2, *p* < 0.001)].

**Figure 3:**
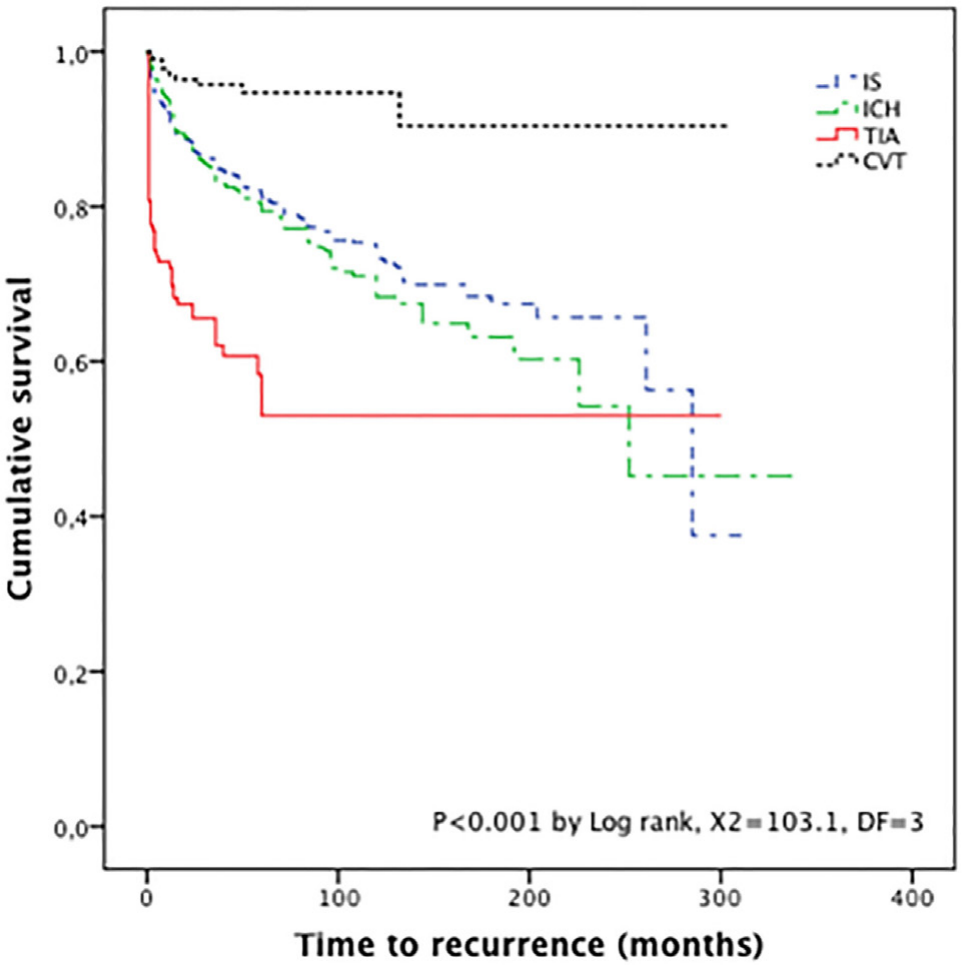
Kaplan–Meier survival curves for recurrence by stroke subtype. Abbreviations: IS, ischemic stroke; ICH, intracranial hemorrhage; TIA, transient ischemic attack; CVT, cerebral venous thrombosis.

Among the 4,224 survivors, we documented 722 recurrent events at the end of follow-up (overall recurrence rate of 17.1%). The recurrence rates for IS, ICH, and CVT patients were: 14.5, 13, and 3.5%, respectively, for the total stroke population, and the median time to recurrence was 12 months (IQR 3–35 months). Fifty-nine TIA patients (35.8% recurrence rate) had a recurrent stroke. Of these patients, 52 (88.1%) had IS, and the remaining patients had another TIA. Seven patients died from recurrence, and the median time to recurrence was 1 month (IQR 1–13 months), with 88% of recurrences occurring during the first month of TIA (52.5% within 1 week). Figure 3 shows the Kaplan–Meier survival curves for recurrence in the stroke subtypes. Diagnosis of TIA was a predictor of recurrence (HR 3.1, 95% CI 2.3–4.0, *p* < 0.001) and HR 2.6 (95% CI 2.1–3.5, *p* < 0.001) after covariate adjustment for sex, age, and risk factors. CVT was a negative predictor of recurrence (HR 0.39 95% CI 0.22–0.70, *p* = 0.002).

## Discussion

This study describes one of the largest hospital-based stroke registries in LA, and in spite of the limitations of single center registries, it shows significant differences with other populations, including a younger age, relatively less hypertension, and larger proportion of CVT. Hypertensive ICH was common and associated with a worse prognosis. A poor functional outcome was observed in most patients and had a high rate of recurrence. Hospital-based stroke registries are widely available in the medical literature (1–4). They provide invaluable information regarding accurate subtypes of stroke and etiology as well as facilitate the follow-up of a large population of patients in very specific geographical and clinical settings. This large hospital-based stroke registry from Mexico shares similarities with other stroke registries, both in findings and potential biases, but it still provides valuable information about patients with stroke in Mexico and throughout LA. For instance, in contradiction with numerous reports, we found that the proportion of females was greater (50%). Previously, only Faulkes et al. (7) had reported 53% of female patients in their series of 1,805 patients. This finding can be attributed to the increased number of CVT patients, since most of these cases were puerperal.

The mean age among our patients was substantially lower compared with that from other published hospital-based registries, which ranged from 62 years in the Ege Registry (4), to 73 years in the Tel Aviv Registry (1). The young puerperal patients in our series in addition to the higher proportion of patients with the UE-NE and ODE subtypes of CI, both of which have mean ages below 40 years, account for the age differences. In addition, this finding may be explained by the characteristics of our institution, in which the criteria for admission of patients treated at our center included a set of younger patients transferred from peripheral hospitals.

Hypertension, although the most common vascular risk factor in our populations, was not within the range of other reported registries, which were as high as 68% (8) and almost invariably above 50%; on the other hand, diabetes was highly prevalent in our patients (18.5%) and was more close to that described in Korean populations (22.4%) (9). We observed higher hypertension in ICH patients, increased smoking habits, and increased prevalence of DM in IS patients as well as a very low prevalence of any vascular risk factor among CVT patients. The distribution of IS subtypes was also different (Table 1) due to the high proportion of ODE and UE patients in our population. This finding may be explained by our hospital being a third level facility that accepts references from other hospitals, which enables an understanding of the characteristics of our patients because once a patient enters the health system, they are more likely to be referred to our hospital when a young age, atypical neurological/neuroimaging findings, or lack of well-known vascular risk factors are present, resulting in an underrepresentation of specific stroke categories (due to rapid lethality or reversible symptoms) and overrepresentation of other factors (nonatherosclerotic vasculopathies or secondary ICH).

A very positive fact regarding our patients is that beyond initial brain imaging, patients were all subsequently examined using a battery of systematic ancillary tests that were aimed at establishing the extent of stroke etiology, thereby reducing the number of patients with unidentified etiology due to an incomplete evaluation. In a Taiwanese registry of patients with CI, this category comprises 29% of 676 UE patients (2), and in other series, the UE proportion ranges from 27% (10) to as low as 1.5% in Italy (11).

Overall mortality was consistent with other available data and the worst prognosis and highest mortality was observed in ICH (12). However, the overall functional outcome observed in our patients was inconsistent with the literature; for instance, in CVT patients, we observed an independency rate at the end of follow-up of 84.9% with mRS ≤3, which was very similar to the 81% independency that was recently reported in this subset of patients (13). Nevertheless, the overall functional outcomes in our series were slightly worse compared to other series (14), with 58.1% (1,964 patients) achieving functional independence (mRs < 3) at the end of follow-up and 41.9% being permanently disabled. Our finding of a mRS of 5 at discharge was a strong predictor of death and explained the minimal proportion of patients in this category at the end of follow-up (1.9%, 63 patients). A higher recurrence rate was observed in our population, and nearly 2 in 10 survivors of stroke patients were readmitted for recurrence within the follow-up time. A higher recurrence rate was observed in ICH patients compared with IS (18 vs. 16.6%). Although heterogeneity in recurrence patterns by geographic region has been recognized (15), we believe that beyond such geographical effects, our high recurrence rate was mostly due to insufficient implementation of secondary prevention interventions as well as to patients who failed to comply with them. Specifically, in hypertension, both problems along with other social and cultural aspects that are present in both in LA and Mexicans living in developed nations (16, 17), have been largely described (18), but there are still areas in which there is much room to improve (19).

Some limitations also should be acknowledged. First, as this is retrospective analysis from a 25 years dataset, the study has the bias of an observational study. Second, as the NINN is a Neurological hospital, this registry reflects the risk factor patterns and clinical course of stroke in a tertiary stroke center in Mexico city. The frequency of some types of stroke could sub- or over-represented; especially uncommon conditions of stroke are referred to our center for subsequent studies, therefore, the extrapolation of our registry becomes difficult to be applied to the rest of the country.

Third, there have been changes in medical practice, including new technical and therapeutic approaches, and clinical evidence guidelines, since past 20 years in the stroke field, therefore, it is difficult to standardize therapeutic options for operative and association analysis at the entire dataset; also as our center is a referral hospital, the acute phase of the index stroke in several cases, has been performed in peripheral hospitals. Such an example is the low IV thrombolysis rate in acute IS (2%), which is explained because the registry only accounts for those patients treated at our center, and many of the included patients are referred from other hospitals; also, the availability of alteplase at the NINN started in 2000.

## Conclusion

This registry adds important data to the understanding of the geographic differences in the stroke subtype distribution and outcomes as well as clearly depicts the risk factor patterns and long-term course of stroke in Mexican patients.

## Ethics Statement

The registry was approved by the “Comité de Bioética (IRB) del Instituto Nacional de Neurologia y Neurocirugía Manuel Velasco Suárez,” since its retrospective nature no informed consent was required.

## Author Contributions

AA, CC-B, and FB conceived and supervised the study. AA, JM-R, and MB drafted the manuscript. JM-R and MB were involved in data analysis and interpretation. The rest of the authors contributed equally to this work, discussed the results and implications, and commented on the manuscript at all stages, and finally approved the final version to be published.

## Conflict of Interest Statement

The authors declare that the research was conducted in the absence of any commercial or financial relationships that could be construed as a potential conflict of interest.
